# Impact of perinatal factors on T cells and transcriptomic changes in preterm infant brain injury

**DOI:** 10.1186/s12974-024-03311-4

**Published:** 2024-11-29

**Authors:** Xiaoli Zhang, Yu Yang, Yiran Xu, Liuji Chen, Ming Niu, Jinjin Zhu, Shan Zhang, Yanan Wu, Bingbing Li, Lingling Zhang, Juan Song, Falin Xu, Dan Bi, Xin Zhao, Changlian Zhu, Xiaoyang Wang

**Affiliations:** 1 Henan Key Laboratory of Child Brain Injury and Henan Pediatric Clinical Research Center, Third Affiliated Hospital and Institute of Neuroscience of Zhengzhou University, Zhengzhou, 450052 China; 2 Department of Pediatrics, Qilu Hospital of Shandong University, Jinan, Shandong China; 3 Department of Imaging, The Third Affiliated Hospital of Zhengzhou University, Zhengzhou, China; 4 Center for Brain Repair and Rehabilitation, Institute of Neuroscience and Physiology, University of Gothenburg, Box 436, Gothenburg, 405 30 Sweden; 5 Center for Perinatal Medicine and Health, Institute of Neuroscience and Physiology, Sahlgrenska Academy, Institute of Clinical Sciences, University of Gothenburg, Box 432, Gothenburg, SE-405 30 Sweden

**Keywords:** Preterm infants, Brain injury, T lymphocytes, Gene expression

## Abstract

**Background:**

T cells have been implicated in various neurological conditions, yet their role in neonatal brain injuries remains unclear. This study aimed to investigate the impact of perinatal factors on frequencies of T cell subsets in preterm infants and to explore the differences in blood genome expression profiles between preterm infants with and without brain injury.

**Materials and methods:**

Three cohorts of preterm infants were used. Blood samples were collected soon after birth for the first cohort and late timepoint for the second and third cohorts. In the first cohort (88 infants), flow cytometry measured the proportions of αβT and γδT cell subsets in peripheral blood, analyzing associations with gestational age, birth weight, sex, delivery type, and maternal conditions. The second cohort focused on the relationship between T cell subsets and brain injury. In the third cohort, transcriptome sequencing identified differentially expressed genes and pathways in infants with brain injury, highlighting immune-related changes.

**Results:**

Infants born at 29–30 weeks or with a birth weight of 1000–1500 g had significantly higher proportions of Vδ2^+^ T cells compared to those born at 30–32 weeks or with a birth weight > 1500 g, while no significant difference was found between infants born at < 29 weeks or with a birth weight < 1000 g. A negative correlation was observed between gestational age and Vδ2^+^ T cell frequency. No significant associations were found between Vδ2^+^ T cell proportions and perinatal factors other than gestational age or brain injury. Blood transcriptome analysis revealed 173 differentially expressed genes, characterized by downregulated interferon signaling and upregulated antimicrobial and neutrophil pathways in infants with brain injury.

**Conclusions:**

Gestational age and birth weight influence Vδ2^+^ T cell proportions in preterm infants, likely reflecting immune maturation. While no direct link to brain injury was found, altered immune pathways suggest potential biomarkers for prognosis, warranting further research into their roles and therapeutic implications in neonatal brain injuries.

**Supplementary Information:**

The online version contains supplementary material available at 10.1186/s12974-024-03311-4.

## Introduction

Preterm birth is a global health issue, responsible for significant neonatal mortality and long-term neurodevelopmental challenges [1, 2]. Infants born before 32 weeks of gestation are particularly vulnerable, with higher risks of brain injuries such as punctate white matter lesions and germinal matrix hemorrhage (GMH). These injuries can have profound effects on cognitive and motor development, creating socioeconomic challenges for infants, families and healthcare systems [3, 4]. Despite extensive research, effective clinical interventions for preventing or mitigating these injuries remain elusive [5], highlighting the urgent need for a deeper understanding of the underlying mechanisms and potential biomarkers of preterm birth related brain injury.

One of the key areas of research in neonatal brain injury is the neuroimmune axis, particularly their role of unconventional T cells like γδT cells in brain homeostasis and injury response [6]. Previous studies have shown altered γδT cell frequencies in neonatal brain injuries, suggesting a potential role for these cells in neonatal brain damage [7–10]. However, the specific influence of perinatal factors on T cell subsets, particularly Vδ2^+^ T cells, in very preterm infants remains largely unexplored.

In this context, immune system development, especially that of T cells, plays a critical role in both brain development and injury response in neonates. γδT cells, which constitute a small but significant proportion of the peripheral T cell population, have been implicated in neuroinflammatory processes in various preclinical models. Within this subset, Vδ2^+^ T cells are of particular interest due to their early appearance during fetal development and their potential cytotoxic and immune-regulatory roles. However, the relationship between Vδ2^+^ T cell frequencies and brain injury in preterm infants has yet to be fully characterized.

In addition to immune cell dynamics, the molecular mechanisms underlying brain injury can be further elucidated through transcriptome analysis. Blood-based transcriptomics provides a window into the gene expression changes associated with immune responses and neural damage in conditions such as neonatal encephalopathy [11–15]. Identifying these transcriptomic alterations could lead to the discovery of novel biomarkers for early diagnosis and prognosis of brain injuries in preterm infants.

The aim of this study was to investigate the impact of maternal and infant-related perinatal variables on the proportions of T cell subsets, particularly Vδ2^+^ T cells, at birth and to assess their association between these immune cells and brain injuries in preterm infants. Additionally, we seek to explore the molecular mechanisms underlying brain injury by analyzing transcriptome profiles in affected infants to identify differentially expressed genes and altered pathways that may contribute to the condition. This combined approach may help uncover potential biomarkers for brain injury and offer insights into the development of targeted therapeutic interventions.

## Methods

### Study design and sample collection

This study was conducted at the Third Affiliated Hospital of Zhengzhou University and involved birth cohorts before 32 weeks of gestation. Participants were recruited based on the following inclusion criteria: preterm birth before 32 weeks of gestation and availability of parental consent for participation. Exclusion criteria included major congenital malformation, genetically determined disease, and/or serious illness.

Peripheral blood samples were collected from three distinct cohorts of preterm infants at different time points and for separate analyses. Cohort 1 included infants from whom blood samples were obtained within three hours of birth, specifically within 0.5 h for 77 infants on non-invasive respiratory support (CPAP) and between 0.5 h and 3 h for 11 infants on mechanical ventilation. Among the 11 infants, three of them received saline and / or the dobutamine for hypotension at birth. A 100 µL sample from this cohort was used for T cell subset analysis. Cohorts 2 and 3 consisted of infants who were diagnosed with brain injuries based on cerebral magnetic resonance imaging (MRI) or ultrasound findings. For Cohort 2, blood samples were collected for flow cytometry analysis, while Cohort 3 samples were collected for transcriptome analysis. Infants in these two cohorts were categorized into the brain injury (BI) group, which included those with significant abnormalities (e.g., encephalomalacia, haemorrhage), and the “no brain injury” (NBI) group, which included infants without significant brain injuries. Blood samples were collected during routine clinical procedures to minimize stress, and all samples were processed within 4 h of collection to ensure cell and RNA integrity. The clinical context, ages, and corrected gestational ages of these infants are provided in Table S1.

Ethical approval was obtained from the Ethics Committees of The Third Affiliated Hospital of Zhengzhou University (ethical no: 2017068), and informed consent was secured from the parents or legal guardians of all participating infants. The study was conducted in accordance with the Declaration of Helsinki, and every effort was made to minimize the stress and discomfort of the infants during blood sampling.

### The timing for head ultrasound and MRI

Head ultrasound examinations were initially performed within the first three days after birth, followed by weekly scans up to four weeks, with increased frequency if abnormality was detected. MRI brain imaging was usually recommended at term-equivalent age for preterm infants with brain injuries or risk factors [16].

### Data collection and variables

Clinical data were collected prospectively during hospitalization. Maternal variables including age, mode of delivery (vaginal or cesarean section), and presence of conditions such as chorioamnionitis, preterm premature rupture of membranes (PPROM), preeclampsia, and reproductive tract infections. Infant variables included gestational age, birth weight, sex, and whether the birth was a single or multiple. The clinical diseases of these infants from cohort 2 and 3 such as sepsis, pneumonia, necrotizing enterocolitis (NEC) and bronchopulmonary dysplasia (BPD) were also collected before analysis.

### Measurement of T lymphocytes subsets by flow cytometry

T cell subsets were analyzed using flow cytometry. Mononuclear cells were isolated from the blood samples and stained within 4 h of collection with a panel of antibodies, including anti-CD3-APC-H7 (clone: SK7), anti‐CD4-BV605(clone: RPA-T4), anti‐CD8-PE-Cy7(clone: RPA-T8), Anti-TCR αβ-FITC (clone: T10B9), anti-TCR γδ-V421(clone: B1), and anti-TCR δ2-PE (clone: B6). All the antibodies were sourced from BD Biosciences. The stained cells were analyzed using an eight-channel flow cytometer (FACS Canto II, Becton Dickinson). Gating strategies were employed to define lymphocyte populations and identify specific T cell subsets, including γδT cells, Vδ2^+^ T cells, and αβT cells. The results were expressed as the percentage of each subset relative to the total T cell population.

### RNA sequencing and transcriptome analysis

For transcriptome analysis, RNA was extracted from the peripheral blood samples using the RNeasy mini kit (Qiagen, Hilden, Germany) according to the manufacturer’s protocol. RNA integrity was assessed using an Agilent Bioanalyzer, and libraries were prepared from total RNA according to manufacturer instructions using the TruSeq Stranded Total RNA Library Prep Kit with Ribo-Zero Gold (Illumina, Cat. No. RS-122-2301). Sequencing was performed on an Illumina platform, generating paired end reads. The transcriptome sequencing data generated in this study available from Bioproject (https://www.ncbi.nlm.nih.gov/bioproject/1154328). The quality of the read sequences (fastq files) was assessed using FastQC (version 0.11.7) and low-quality reads (< Q20) and trimmed reads with adaptor sequences shorter than 50 bp were removed using Cutadapt (version 1.16). Differentially expressed genes between infants with/without brain injury were identified using the deseq2 packages in R (version 4.1.0). Genes with an adjusted p-value < 0.05 were considered significant. Gene set enrichment analysis (GSEA) [17] was performed to identify enriched pathways and biological processes, with reference to MSigDB Collections (https://www.gsea-msigdb.org/gsea/msigdb/collections.jsp). The ggplot2 package was used to visualize the results of the enrichment analysis.

### Evaluation of immune cell types

The single-sample Gene Set Enrichment Analysis (ssGSEA) algorithm was utilized to deconvolute immune cell types from the transcriptome data dataset comprising markers for 24 immune cell types was used to identify and quantify the presence of different immune cell types in the blood samples [18]. Box plot was used for visualization the distribution of immune cell types between the infants with/without brain injury groups.

### Statistical analysis

Data were analyzed using SPSS21.0 (IBM, USA). Normality of the data was assessed using the Shapiro–Wilk test before subsequent statistical analysis, and homogeneity of variances was evaluated with Levene’s test. To compare two groups, either a two-tailed unpaired t-test or Mann–Whitney U-test or chi-squared test was used. To compare multiple groups, one-way ANOVA or Kruskal-Wallis tests were used, depending on the normality of the data distribution. Correlations between variables were assessed using linear regression analysis. Gene expressions data (FPKM) produced by RNAseq were analyzed using the DEseq2 software package, which is designed for normalization, visualization, and differential analysis of high-dimensional count data. A *p*-values or adjusted *p*-values < 0.05 were considered statistically significant.

## Results

### Impact of perinatal factors on T cell subsets

**Fig. 1:**
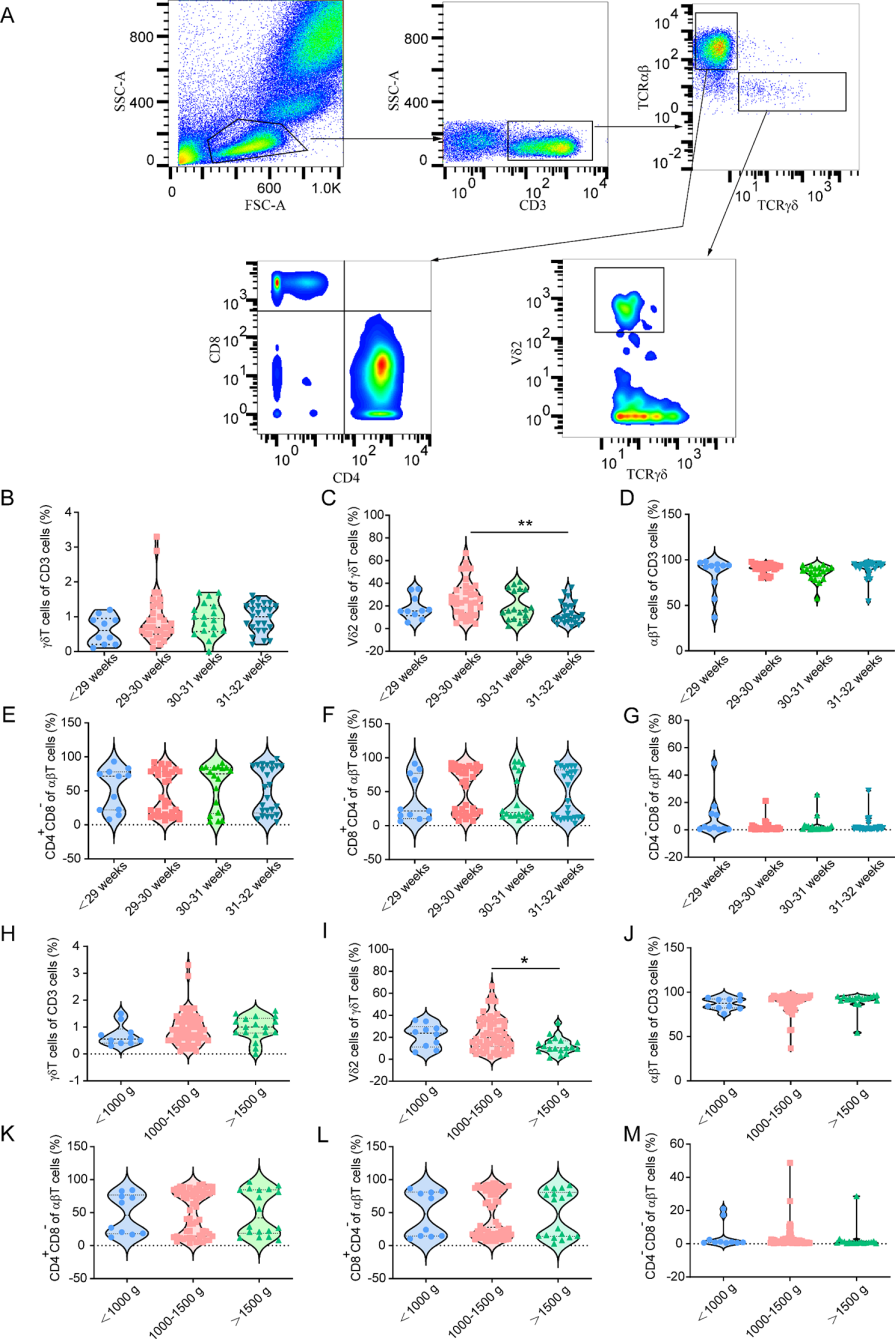
Gating strategy and proportions of T cell subsets in peripheral blood by gestational age and birth weight in preterm infants. **A**. Flow cytometry gating strategy used to define lymphocyte populations, including αβT cells, γδT cells, and Vδ2^+^ T cells. **B-G**. Proportions of T cell subsets by gestational age: **B**. γδT cells, **C**. Vδ2^+^ T cells, **D**. αβT cells, **E**. CD4^+^CD8^−^ cells, **F**. CD8^+^CD4^−^ cells, and **G**. CD4^−^CD8^−^ cells. **H-M**. Proportions of T cell subsets by birth weight: **H**. γδT cells, **I**. Vδ2^+^ T cells, **J**. αβT cells, **K**. CD4^+^CD8^−^ cells, **L**. CD8^+^CD4^−^ cells, and **M**. CD4^−^CD8^−^ cells. One-way ANOVA or Kruskal-Wallis test was used for multiple comparisons. B-G: *n* = 11–34/group, H-M: *n* = 10–60/group; *adjusted *p* < 0.05, ** adjusted *p* < 0.01

Flow cytometry analysis was performed on 88 periphery blood samples from preterm infants to assess the impact of perinatal factors on T cell subsets. The gating strategy used to define lymphocyte populations and T cell subsets is illustrated in Fig. 1A. The analysis revealed that gestational age had no effect on γδT cells (Fig. 1B, *p* > 0.05). The proportion of Vδ2^+^ T cells among γδT cells was significantly higher in preterm infants born at 29–30 weeks of gestation compared to those born at less than 29 weeks or at 30–32 weeks (H = 22.94, *p* = 0.004, Fig. 1C). γδT cells, αβT cells, CD4^+^CD8^−^ subset, CD8^+^CD4^−^ subset and CD4^−^CD8^−^ subset had no difference among groups of different gestational ages (Fig. 1D-G, *p* > 0.05). Among the birth weight groups of < 1000 g, 1000–1500 g and > 1500 g, there was no difference of γδT cells (Fig. 1H, *p* > 0.05). Infants with a birth weight of 1000–1500 g had a significantly higher proportion of Vδ2^+^ T cells compared to those with a birth weight less than 1000 g or greater than 1500 g, as shown in Fig. 1I (H = 17.53, *p* = 0.032). αβT cells, CD4^+^CD8^−^ subset, CD8^+^CD4^−^ subset and CD4^−^CD8^−^ subset had no difference among groups of body weight at birth (Fig. 1J-M, *p* > 0.05).

**Fig. 2:**
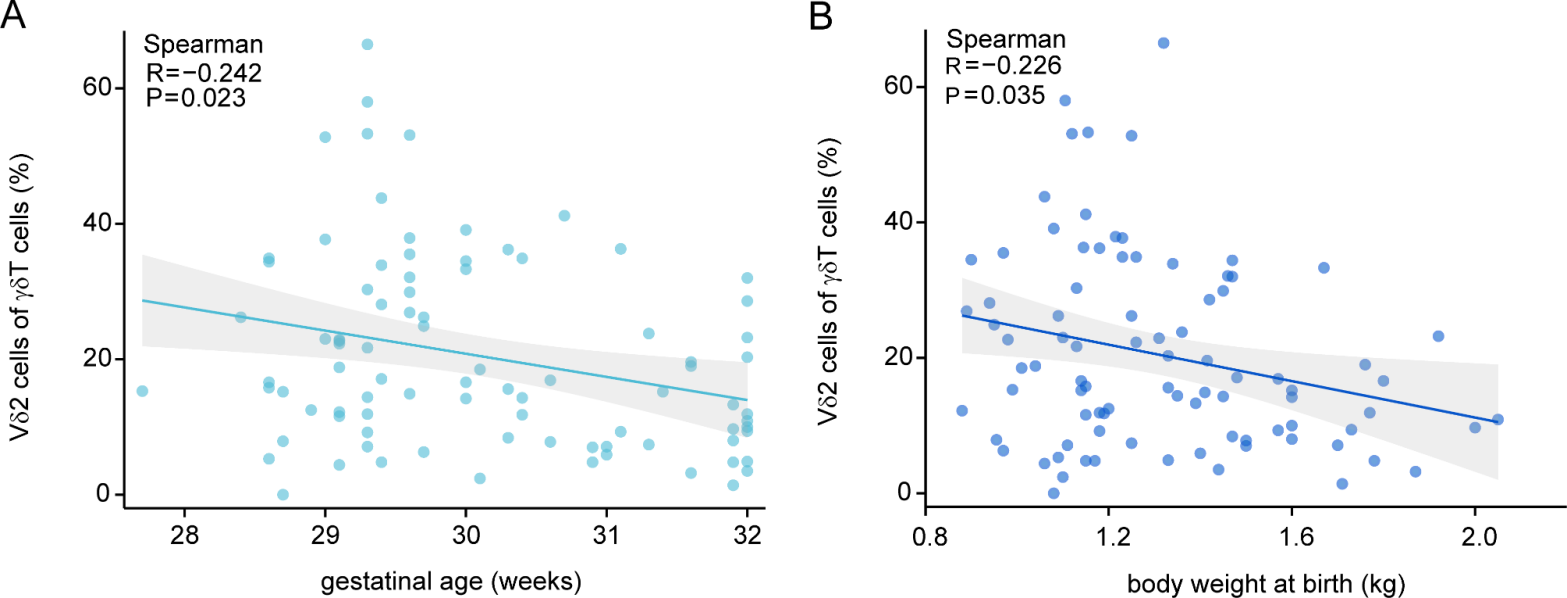
Correlation between gestational age, birth weight, and Vδ2^+^T cell proportions. **A**. Scatterplots illustrating the negative correlation between gestational age and the percentage of Vδ2^+^ T cells among γδT cells (*r* = -0.242, *p* = 0.023). **B**. Scatterplots illustrating the negative correlation between birth weight and the percentage of Vδ2^+^ T cells (*r* = -0.226, *p* = 0.035). The upper and lower reference ranges of the area under the curve are highlighted in grey

To further investigate the relationship between gestational age, birth weight, and Vδ2^**+**^ T cell proportions, a linear correlation analysis was conducted. Results showed a significant negative correlation between gestational age and Vδ2^+^ T cell proportions (*r* = -0.242, *p* = 0.023, Fig. 2A), as well as between birth weight and Vδ2^+^ T cell proportions (*r* = -0.226, *p* = 0.035, Fig. 2B).

Other perinatal factors, including sex, single or multiple birth, maternal age, mode of delivery, chorioamnionitis, PPROM, preeclampsia, and reproductive tract infections, did not show significant associations with the proportions of Vδ2^+^ T cells or other T cell subsets (γδT cells, αβT cells, CD4^+^CD8^−^ CD8^+^CD4^−^ and CD4^−^CD8^−^) in peripheral blood (Table 1).

**Table 1:** Cohort characteristics of infants with T cell subsets measurements at birth

*n* = 88	γδT cells (%)	Vδ2^+^T (%)	αβT (%)	CD4^+^CD8^−^(%)	CD8^+^CD4^−^(%)	CD4^−^CD8^−^(%)
Sex						
male (*n* = 40)	0.89 ± 0.64	22.98 ± 15.06	87.37 ± 12.29	0.12 ± 0.09	47.58 ± 33.15	4.08 ± 8.89
female (*n* = 48)	0.89 ± 0.44	17.33 ± 12.73	90.38 ± 6.24	0.15 ± 0.29	44.75 ± 34.78	2.33 ± 3.92
Single/multiple						
Single (*n* = 59)	0.85 ± 0.48	20.12 ± 13.19	88.76 ± 9.43	0.14 ± 0.25	50.43 ± 33.40	2.76 ± 5.36
Multiple (*n* = 29)	0.98 ± 0.69	21.01 ± 16.43	88.68 ± 11.43	0.12 ± 0.09	37.88 ± 33.41	4.37 ± 9.76
Maternal age						
<35 years (*n* = 60)	0.93 ± 0.56	20.42 ± 14.77	88.44 ± 10.05	0.12 ± 0.09	41.82 ± 34.09	3.58 ± 7.82
≥ 35 years (*n* = 28)	0.81 ± 0.55	20.39 ± 13.35	89.37 ± 10.26	0.17 ± 0.35	55.88 ± 31.45	2.66 ± 5.30
Delivery mode						
Vaginal (*n* = 19)	0.84 ± 0.43	18.98 ± 11.41	90.32 ± 4.70	0.11 ± 0.05	51.39 ± 34.46	2.29 ± 4.76
Cesarean section (*n* = 69)	0.91 ± 0.59	20.81 ± 14.99	88.31 ± 11.09	0.14 ± 0.23	44.89 ± 33.66	3.56 ± 7.62
Chorioamnionitis						
No (*n* = 83)	0.90 ± 0.56	20.33 ± 14.42	88.37 ± 10.24	0.14 ± 0.21	44.51 ± 33.24	3.45 ± 7.28
Yes (*n* = 5)	0.71 ± 0.42	21.75 ± 12.29	94.84 ± 2.26	0.13 ± 0.06	75.94 ± 30.57	0.58 ± 0.10
PPROM						
no (*n* = 61)	0.82 ± 0.48	19.98 ± 12.49	86.89 ± 11.48	0.14 ± 0.24	48.21 ± 32.94	4.16 ± 8.27
<18 h (*n* = 8)	0.85 ± 0.33	11.67 ± 6.40	92.41 ± 3.12	0.09 ± 0.06	53.02 ± 35.85	0.88 ± 0.65
≥ 18 h (*n* = 19)	1.14 ± 0.77	25.47 ± 19.60	93.12 ± 2.92	0.12 ± 0.07	37.33 ± 35.59	1.51 ± 2.64
Preeclampsia						
no (*n* = 49)	0.93 ± 0.61	21.47 ± 14.71	90.29 ± 9.28	0.13 ± 0.08	45.74 ± 35.43	3.06 ± 7.79
yes (*n* = 39)	0.86 ± 0.49	19.09 ± 13.72	86.78 ± 10.78	0.15 ± 0.29	47.00 ± 31.93	3.57 ± 6.21
Reproductive tract infection						
no (*n* = 80)	0.90 ± 0.57	20.40 ± 14.15	88.27 ± 10.40	0.14 ± 0.21	45.03 ± 33.43	3.54 ± 7.40
yes (*n* = 8)	0.80 ± 0.47	20.51 ± 16.35	93.44 ± 3.37	0.11 ± 0.05	58.96 ± 36.49	0.75 ± 0.66

Overall, while most T cell subsets remain unaffected by perinatal factors, Vδ2^+^ T cells exhibit specific associations with gestational age and birth weight, suggesting a unique role or developmental pattern for this subset in preterm infants.

### T cell subsets in brain injuries

**Fig. 3:**
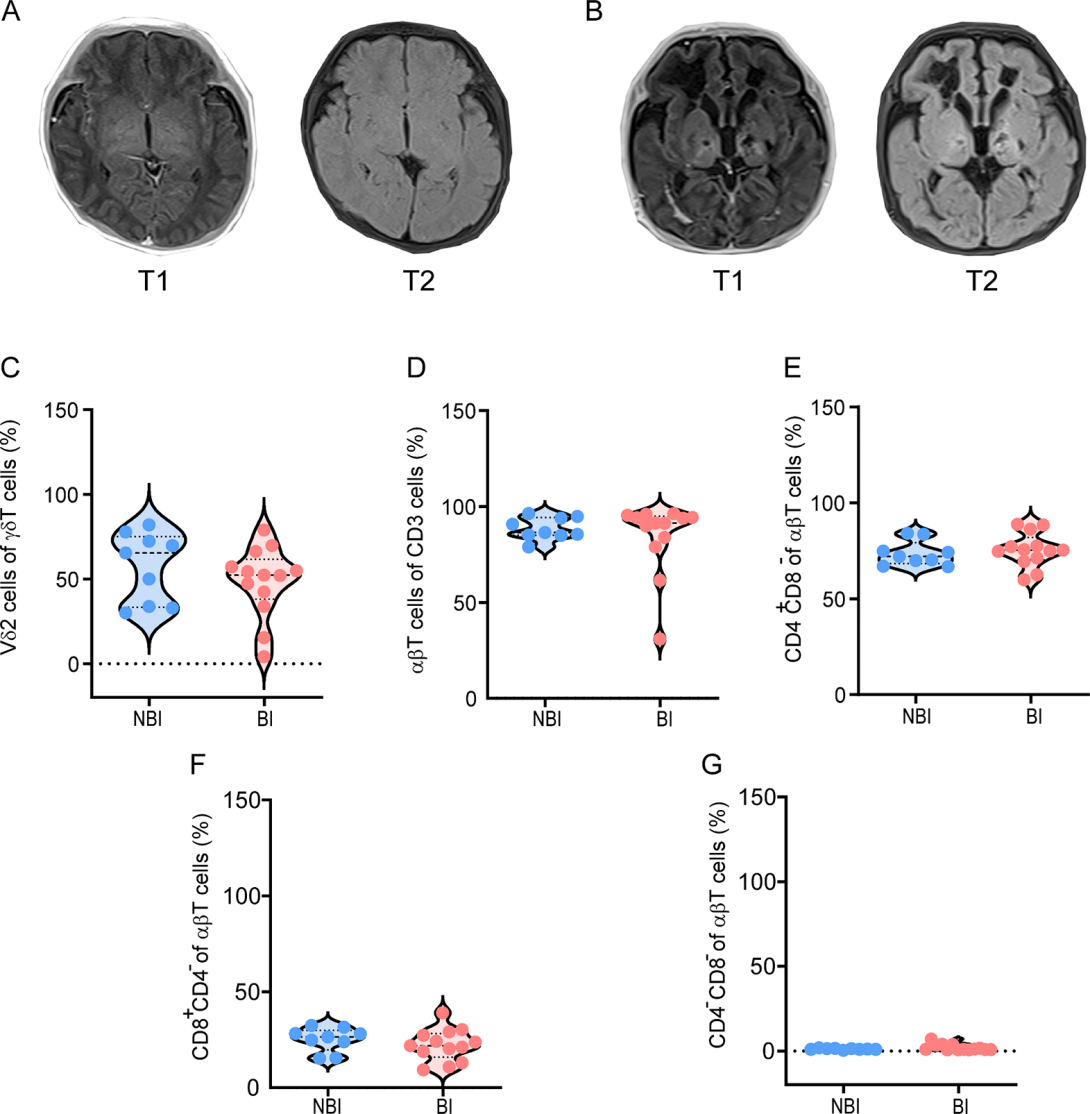
Comparison of T cell subsets in infants with and without brain injury. **A**. Cerebral MRI T1 and T2 images from infants without brain injury (case no: NBI-9). **B**. Cerebral MRI T1 and T2 images from an infant with brain injury (case no: BI-8). **C-G**. Proportion of T cell subsets in infants with/without brain injury: **C**. Vδ2^+^ T cells, **D**. αβT cells, **E**. CD4^+^CD8^−^ cells, **F**. CD8^+^CD4^−^ cells, and **G**. CD4^−^CD8^−^ cells. t-test or Mann–Whitney U-test for comparison. *n* = 9–13/group

Next, we analyzed whether preterm infants with brain injury exhibit altered proportions of peripheral blood T lymphocytes. We initially analyzed the demographic and clinical characteristics of infants with and without brain injury in relation to flow cytometry testing, as presented in Table 2. There were no statistically significant differences between the NBI and BI groups in terms of infant characteristics such as sex and birth weight, nor in maternal factors including age, delivery mode, chorioamnionitis, PPROM, preeclampsia, and reproductive tract infection (*p* > 0.05). Similarly, the incidence of conditions such as sepsis, pneumonia, necrotizing enterocolitis (NEC), and bronchopulmonary dysplasia (BPD) was comparable between the BI and NBI groups (*p* > 0.05). Additionally, the mean corrected gestational age (GA), age at examination, and C-reactive protein (CRP) levels did not differ significantly between the two groups (*p* > 0.05). Representative T1 and T2 MRI images from infants with and without brain injury are shown in Fig. 3A and B, respectively. No significant differences were observed in the proportions of Vδ2^**+**^ T cells, αβT cells, CD4^+^CD8^−^ CD8^+^CD4^−^ and CD4^−^CD8^−^ subsets between infants with and without brain injury (*p* > 0.05, Fig. 3C and G). This indicates that, at least in terms of peripheral blood T cell composition, brain injury may not be associated with detectable proportion changes in these specific immune cell populations.

**Table 2:** Demographic and clinical characteristics of infants with/without brain injury for T cells subsets analysis

Variables	NBI group (*n* = 9)	BI group (*n* = 13)	*p*
Male	9(0)	13(0)	/
Body weight (g) at birth	1196 ± 219	1323 ± 345	0.35
Gestational age (weeks)	28.89 ± 1.53	29.12 ± 1.80	0.76
Maternal age	30.44 ± 3.81	30.00 ± 3.67	0.78
Cesarean section	8(1)	10(3)	0.47
Chorioamnionitis	0(9)	2(11)	0.22
PPROM	2(7)	5(8)	0.42
Preeclampsia	2(7)	2(11)	0.68
Reproductive tract infection	0(9)	2(11)	0.22
Pneumonia	3(6)	8(5)	0.19
Sepsis	1(8)	4(9)	0.28
BPD	5(4)	4(9)	0.24
NEC	1(8)	4(9)	0.28
CRP (mg/L)	1.89 ± 2.49	2.66 ± 4.22	0.63
Mean corrected GA at examination	34.69 ± 2.15	35.41 ± 2.33	0.48
Ages at examination (days)	40.77 ± 15.05	43.92 ± 16.04	0.65

### Transcriptome analysis and pathway enrichment in infants with brain injury

**Fig. 4:**
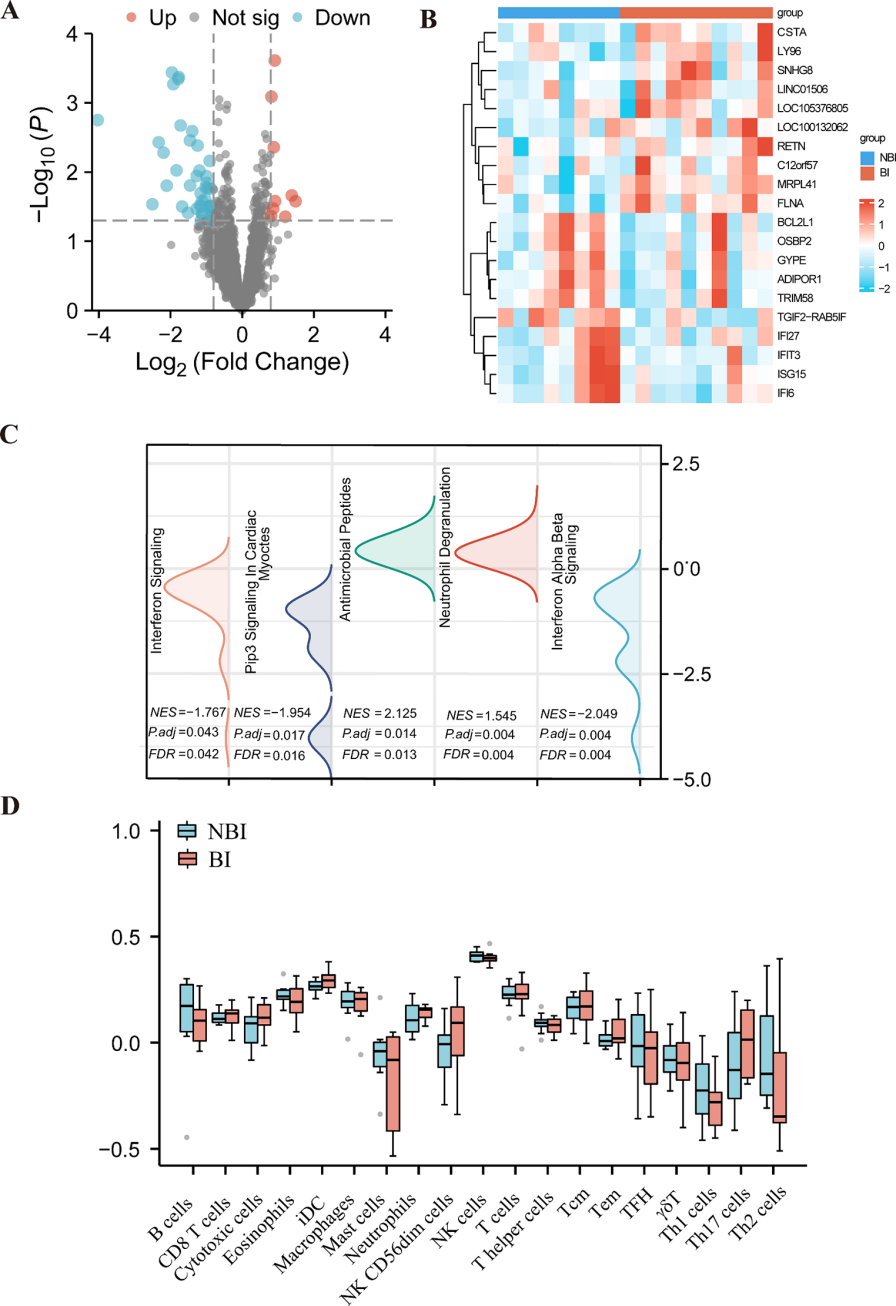
Differential gene expression and pathway analysis in infants with brain injury. Volcano plot showing differential gene expression between infants with /without brain injury. The screening threshold is set at |FC|>0.80, where red dots represent upregulated genes, and blue dots represent downregulated genes. **B**. Heatmap displaying the top ten upregulated and downregulated genes. **C**. GSEA results showing up- and down- regulating pathways. **D**. ssGSEA showing differences in the composition of 19 immune cell types between infants with/without brain injury. *n* = 8–10/group

To further investigate potential molecular mechanisms underlying brain injury, we performed RNA sequencing (RNA-seq) on peripheral whole blood samples from infants with/without brain injury. The demographic and clinical characteristics of these infants are presented in Table 3. No significant differences were observed between the NBI and BI groups in terms of infant characteristics such as sex and birth weight, nor in maternal factors including age, delivery mode, chorioamnionitis, PPROM, preeclampsia, and reproductive tract infection (*p* > 0.05). Similarly, the prevalence of conditions such as sepsis, pneumonia, NEC, and BPD did not differ significantly between the two groups (*p* > 0.05). Mean corrected GA, age at examination, and CRP levels were also comparable (*p* > 0.05). Differential gene expression analysis identified 173 differentially expressed genes between infants with/without brain injury, including 68 upregulated genes and 105 downregulated genes (*p* < 0.05, Fig. 4A and Table S2). The top ten upregulated and downregulated genes are listed in Fig. 4B and Table S3- S4. The top ten upregulated genes in infants with brain injury include genes involved in immune responses, such as RETN (antimicrobial defense) and LY96 (immune signaling via TLR4). The downregulated genes include those linked to apoptosis and immune responses, such as IFI27, IFI6, and ISG15, alongside genes involved in lipid metabolism (OSBP2) and apoptosis regulation (BCL2L1).

**Table 3:** Demographic and clinical characteristics of infants with/without brain injury for transcriptome analysis

Variables	NBI group (*n* = 8)	BI group (*n* = 9)	*p*
Male	5(3)	5(4)	0.77
Body weight (g) at birth	1258.0 ± 217.6	1461.2 ± 442.9	0.26
Gestational age (weeks)	29.91 ± 1.74	29.92 ± 1.95	0.99
Maternal age	30.13 ± 1.46	32.00 ± 4.66	0.29
Cesarean section	5(3)	6(3)	0.85
Chorioamnionitis	1(7)	0(9)	0.86
PPROM	3(5)	4(5)	0.77
Preeclampsia	2(6)	1(8)	0.27
Reproductive tract infection	1(7)	1(8)	0.93
Pneumonia	3(5)	3(6)	0.85
Sepsis	1(7)	2(7)	0.60
BPD	2(6)	3(6)	0.71
NEC	2(6)	1(8)	0.45
CRP (mg/L)	5.34 ± 6.34	4.60 ± 4.36	0.78
Mean corrected GA at examination	35.17 ± 0.96	35.39 ± 1.36	0.71
Ages at examination (days)	37.78 ± 12.07	38.22 ± 19.78	0.96

GSEA revealed 12 enriched gene sets for specific biological processes (Table S5) and 6 meaningful immune-related signaling pathways. Among the upregulated processes in brain injury group were antimicrobial peptides (NES = 2.125, FDR = 0.013) and neutrophil degranulation (NES = 1.545, FDR = 0.004). On the other hand, several processes were downregulated, including interferon alpha/beta signaling (NES = -2.049, FDR = 0.004), Pip3 signaling in cardiac myocytes (NES = -1.954, FDR = 0.042) and interferon signaling (NES = -1.767, FDR = 0.042) (Fig. 4C). These results, consistent with the top up- and downregulated genes, suggest that alterations in immune signaling pathways may play a role in the pathogenesis of brain injury in preterm infants.

The immune cell profiles, as assessed by ssGSEA algorithm, did not reveal significant differences in the distribution of 19 immune cell types between infants with/without brain injury (Fig. 4D and Table S6). This suggests that brain injury in preterm infants is linked to specific immune pathway alterations, including increased inflammation and reduced antiviral signalling, which may contribute to the pathogenesis of brain injury or reflect the body’s response to it.

## Discussion

This study aimed to elucidate the impact of perinatal factors on the proportions of different T cell subsets in the peripheral blood of preterm infants and explore their potential association with brain injury. Our findings indicate that most T cell subsets, including γδT cells, αβT cells, and various CD4^+^ and CD8^+^ subpopulations, were not significantly influenced by gestational age, birth weight, or other perinatal factors. These findings suggest that T cell distribution in preterm infants is relatively stable and may be less susceptible to perinatal variations than anticipated. Interestingly, we found distinct associations between Vδ2^+^ T cell proportions and both gestational age and birth weight. Specifically, we observed the higher proportions of Vδ2^+^ T cells in infants born at 29–30 weeks gestation and with a birth weight of 1000–1500 g compared to those born later or with larger birth weights. These associations suggest a potential role for Vδ2^+^ T cells in immune maturation during this critical developmental window. However, no significant link between Vδ2^+^ T cells and brain injury was identified, pointing to the possibility that other immune pathways may play a more prominent role in the pathogenesis of neonatal brain injury.

The higher proportions of Vδ2^+^ T cells were observed in infants born at 29–30 weeks gestation and those with a birth weight between 1000 and 1500 g. The significant negative correlation between Vδ2^+^ T cell proportions and both gestational age and birth weight suggests that these cells are more prominent during a critical developmental window. The absence of significant associations between Vδ2^+^ T cell proportions and other perinatal factors, such as sex, type of birth, and maternal conditions, further emphasizes the specificity of the relationship between Vδ2^+^ T cells and the developmental parameters of gestational age and birth weight. Indeed, the development of γδT cells, including the Vδ2^+^ subset, is closely tied to fetal maturation [19, 20], and that γδT cells are among the earliest T cells to emerge as early as eight weeks of gestation [21–24].

The immune system, particularly T cells, plays a crucial role in neonatal brain development and injury response. γδT cells, a subset of T cells constituting approximately up to 5% of total human peripheral T cells and even higher in children, are a crucial for immune surveillance and regulation [25–28]. Notably, preclinical studies have demonstrated that γδT cells contribute to brain injury in various animal models, suggesting their involvement in neuroinflammatory processes [29–32]. Within the γδT cell population, Vδ2^**+**^ T cells are of particular interest due to their early presence in human fetal development and their potent immune functions. These cells typically co-express Vδ9 TCR chain, have been shown to expand and acquire cytotoxic capabilities shortly after birth [27, 33, 34]. Vδ2^**+**^ T cells have been linked to various immune responses, including cytokine production and adaptive immunity enhancement [35, 36].

In our previous studies, significant populations of γδT cells were identified in post-mortem brain tissues from preterm human infants and decreased frequency of γδ T cells in periphery blood in very preterm infant with periventricular leukomalacia (PVL) [9, 29]. In another report, it was also found increased frequency of Vδ2^+^ T cells in perinatal blood in term infants affected by neonatal encephalopathy [8]. There was more frequent presence of γδT cells in periphery blood at two different time points in extremely preterm infants with brain haemorrhage and PVL [10]. Together, these findings indicate a potential involvement of γδT cells, particularly Vδ2^+^ T cells, in the immune response associated with brain injuries in preterm infants. The observed variations in γδT cell populations in both brain tissues and peripheral blood suggest that these cells may play a role in the pathological processes or the body’s response to conditions such as PVL, neonatal encephalopathy, and brain haemorrhage. Further research is needed to clarify whether these changes are a cause of, or a response to, brain injury in preterm infants. To note, the absence of a direct relationship between Vδ2^+^ T cells and brain injuries in this study contrasts with some preclinical findings that have implicated γδT cells in the pathogenesis of neonatal brain injury [29, 30]. This discrepancy may be due to differences between human clinical conditions and animal models, where the mechanisms of injury and immune response could vary significantly [37, 38]. It is also possible that other subsets of immune cells or distinct pathways are more critical in the development of brain injury in preterm infants, necessitating further investigation. Altogether, it highlights the complexity of the immune response in preterm infants and suggests that further investigation is needed to fully understand the role of Vδ2^+^ T cells in the context of brain injury.

Our transcriptome analysis revealed significant alterations in immune-related pathways in infants with brain injury, notably the upregulation of antimicrobial peptides and neutrophil degranulation, alongside the downregulation of interferon alpha/beta signaling. These findings suggest that brain injury in preterm infants may be associated with a dysregulated immune response, potentially contributing to the exacerbation of brain injury [39, 40]. Conversely, downregulation of interferon alpha/beta signalling and interferon signalling pathways might indicate a compromised ability to mount an effective antiviral response [41], which could leave the developing brain vulnerable to viral infections or other inflammatory triggers. Despite these changes, the overall distribution of immune cell types in peripheral blood remained stable, implying that the functional activity of immune cells, rather than their population sizes, is altered in brain injury.

Our study provides valuable insights into the immune characteristics of preterm infants, though several limitations should be noted. The sample size, while sufficient for some analyses, may have limited our ability to detect subtle interactions among perinatal factors, T cell subsets, and immune responses, particularly regarding infections. A larger sample in future studies could enable a more detailed analysis of these associations. The cross-sectional design also restricts causal inferences, as we assessed associations at only a single time point. Longitudinal studies would offer a clearer view of immune development and its relationship to brain injury. Small subgroup sizes, with only 1–2 cases in some groups, limited detailed analysis of confounding factors like sepsis, NEC, and pneumonia in BI and NBI groups. However, Tables 2 and 3 show no significant baseline differences, indicating that these factors likely did not affect T cell outcomes. Lastly, while flow cytometry was robust, it may not fully capture complex immune interactions within brain tissue. Advanced methods could enhance future research on these processes.

In summary, this study highlights the impact of gestational age and birth weight on Vδ2^+^ T cell proportions in very preterm infants, though no direct link between Vδ2^+^ T cells and brain injuries was found. The transcriptomic alterations observed in infants with brain injury suggest that potential immune dysregulation may play a role in the pathogenesis of brain injury, warranting further investigation into these immune pathways. Our findings contribute to the understanding of immune development in preterm infants and point toward new directions for research aim at improving outcomes for this high-risk population.

## Electronic supplementary material

Below is the link to the electronic supplementary material.

Table S2

Table S3-S4

 Table S5

 Table S6

Table S1

## Data Availability

No datasets were generated or analysed during the current study.
