# Supplementary material for: Impact of perinatal factors on T cells and transcriptomic changes in preterm infant brain injury
Source: J Neuroinflammation. 2024 Nov 29;21:310. doi: 10.1186/s12974-024-03311-4 (PMC11607874; doi:10.1186/s12974-024-03311-4)
Supplement: Supplementary file 2 — Table S3-S4 [file 12974_2024_3311_MOESM2_ESM.docx]

**Table S3 The top ten upregulated genes in brain injury group compared to no major brain injury group**

| Gene | Gene description | FC | P-value | Function |
| --- | --- | --- | --- | --- |
| RETN | Resistin | 1.492 | 0.0264 | Involved in regulating insulin sensitivity, inflammation, and antimicrobial defense. |
| LINC01506 | long intergenic non-protein coding RNA 1506 | 1.383 | 0.0217 | Non-protein coding RNA |
| LY96 | Lymphocyte Antigen 96 | 1.202 | 0.0441 | Enhances the response of TLR4 to bacterial lipopolysaccharides, playing a key role in the innate immune response to infection |
| LOC100132062 | Novel Transcript | 0.972 | 0.0279 | Non-protein coding RNA |
| LOC105376805 | Novel Transcript | 0.938 | 0.0485 | Non-protein coding RNA |
| CSTA | Cystatin A | 0.916 | 0.0263 | Encodes cystatin A, a protease inhibitor that protects cells from proteolytic damage, particularly in the skin and epithelial tissues |
| MRPL41 | Mitochondrial Ribosomal Protein L41 | 0.909 | 0.00024 | Ribosome components and involved in cell cycle, apoptosis, cell survival |
| SNHG8 | small nucleolar RNA host gene 8 | 0.881 | 0.00438 | Non-protein coding RNA |
| C12orf57 | Chromosome 12 Open Reading Frame 57 | 0.865 | 0.0336 | Associated with intellectual disability and developmental disorders, including Temtamy syndrome, due to its role in corpus callosum development. |
| FLNA | Filamin A | 0.817 | 0.000815 | Crucial for actin crosslinking, cell structure, and development |

**Table S4 The top ten downregulated genes in brain injury group compared to no major brain injury group**

| Gene | Gene description | FC | P-value | Function |
| --- | --- | --- | --- | --- |
| IFI27 | Interferon Alpha Inducible Protein 27 | -4.035 | 0.00178 | Involved in apoptosis and innate immune response |
| TGIF2-RAB5IF | Read-through transcription between the neighboring TGIF2 and C20orf24 | -2.508 | 0.0292 | Encodes a fusion protein related to cellular signaling pathways |
| IFI6 | Interferon Alpha Inducible Protein 6 | -2.332 | 0.00371 | Involved in apoptosis and immune response to viral infections |
| ISG15 | ISG15 ubiquitin like modifier | -2.197 | 0.00523 | Ubiquitin-like protein involved in interferon-mediated antiviral defense |
| IFIT3 | Interferon induced protein with tetratricopeptide repeats 3 | -2.018 | 0.0157 | Involved in negative regulation of apoptotic process; negative regulation of cell population proliferation; and response to virus |
| OSBP2 | Oxysterol binding protein 2 | -1.963 | 0.000364 | Binds oxysterols, involved in lipid metabolism and cytotoxicity inhibition |
| BCL2L1 | BCL2 like 1 | -1.924 | 0.000538 | Anti-apoptotic regulator, part of the BCL-2 family |
| GYPE | Glycophorin E | -1.835 | 0.00945 | Erythrocyte membrane protein, involved in blood group antigen expression |
| TRIM58 | Tripartite motif containing 58 | -1.785 | 0.000453 | Involved in erythropoiesis and ubiquitin protein ligase activity |
| ADIPOR1 | Adiponectin Receptor 1 | -1.770 | 0.00042 | Regulates glucose levels and lipid metabolism |
