# Supplementary material for: Impact of perinatal factors on T cells and transcriptomic changes in preterm infant brain injury
Source: J Neuroinflammation. 2024 Nov 29;21:310. doi: 10.1186/s12974-024-03311-4 (PMC11607874; doi:10.1186/s12974-024-03311-4)
Supplement: Supplementary file 5 — Table S1 [file 12974_2024_3311_MOESM5_ESM.docx]

**Table S1. Demographic and clinical characteristics of infants with/without brain injury**

| **Group and case** | **Sex** | **GA at birth**  **(gestational weeks +days)** | **Birth weight(g)** | **Report from cerebral**  **MRI or ultrasound** | **Clinical context and diseases** | **Flow**  **cytometry** | **RNA sequencing** | **Timing for 2^nd^ blood samples** | | **CRP**  **(mg/L)** |
| --- | --- | --- | --- | --- | --- | --- | --- | --- | --- | --- |
|  |  |  |  |  |  |  |  | **Age**  **(days)** | **Corrected**  **GA(weeks+days)** |  |
| NBI-1 | M | 28+2 | 1320 | Unremarkable. | Triplet; RDS; BPD; HB. | Yes | No | 26 | 32+0 | 0.20 |
| NBI-2 | M | 28+2 | 1230 | Unremarkable. | Triplet; RDS; Pulmonary hemorrhage; Pneumonia. | Yes | No | 26 | 32+0 | 0.24 |
| NBI-3 | M | 28+1 | 1100 | Bilateral temporal subarachnoid space became slightly wider. | Preeclampsia and Gestational diabetes;  RDS; Pneumonia; BPD; Pneumothorax. | Yes | No | 30 | 32+3 | 0.47 |
| NBI-4 | M | 29+2 | 860 | Abnormal signals in the wall of the lateral ventricle on both sides indicated a small amount of hemorrhage in the ependymal of the lateral ventricle. Bilateral middle ear mastoid effusion or delayed gasification. | Twin-to-twin transfusion syndrome; RDS; HB; Neonatal asphyxia. | Yes | No | 61 | 38+0 | 0.26 |
| NBI-5 | M | 27+3 | 1080 | Bilateral frontotemporal subarachnoid space was slightly wider. | PPROM-5 days; RDS; HB; BPD. | Yes | No | 55 | 35+2 | 3.12 |
| NBI-6 | M | 30+6 | 1610 | Unremarkable. | Gestational diabetes; PPROM-5h; RDS; HB. | Yes | Yes | 26 | 34+4 | 0.73 |
| NBI-7 | M | 30+1 | 1370 | Unremarkable. | Gestational hypertension and preeclampsia; RDS; NEC; BPD; HB; Pneumonia. | Yes | Yes | 47 | 36+5 | 0.15 |
| NBI-8 | M | 26+4 | 900 | Bilateral frontotemporal subarachnoid space became slightly wider; Bilateral mastoid effusion; The signal of posterior pituitary height was not clear. | Threatened preterm labor; RDS; BPD; Sepsis; Hemolytic disease of the newborn (ABO); Pneumonia. | Yes | Yes | 60 | 35+1 | 5.22 |
| NBI-9 | M | 31+0 | 1200 | Unremarkable; Middle ear mastoid fluid or incomplete gasification. | Preeclampsia; fetal distress in uterus; RDS; HB; Pneumonia. | Yes | Yes | 36 | 36+1 | 6.64 |
| NBI-10 | M | 31+6 | 1210 | Unremarkable. | PPROM-3 days; RDS; HB; Neonatal infection. | No | Yes | 35 | 35+6 | 11.6 |
| NBI-11 | F | 28+0 | 1264 | Unremarkable. | Gestational diabetes; RDS; Neonatal infection; NEC. | No | Yes | 45 | 34+3 | 0.42 |
| NBI-12 | F | 30+3 | 1080 | Unremarkable. | Chronic hypertension and preeclampsia; RDS; Neonatal asphyxia; HB. | No | Yes | 25 | 34+0 | 0.56 |
| NBI-13 | F | 30+3 | 1430 | Unremarkable; Bilateral anterior temporal and subarachnoid space was slightly wider; Bilateral middle ear mastoid effusion or incomplete gasification. | PPROM-25 days; Mycoplasma infection in genital tract; RDS; Neonatal infection; Neonatal asphyxia; Pneumonia. | No | Yes | 29 | 34+4 | 17.4 |
| BI-1 | M | 31+6 | 1540 | Encephalomalacia around lateral ventricles. | Fetal distress in uterus; RDS; Bronchopneumonia; Malrotation of intestine | Yes | No | 59 | 40+2 | 0.96 |
| BI-2 | M | 28+2 | 880 | Encephalomalacia in parietal and occipital lobe; The development of white matter of brain was delayed. | Preeclampsia; fetal distress in uterus? RDS; HB; Neonatal asphyxia; Brain hemorrhage and hydrocephalus; Sepsis;  Pneumonia. | Yes | No | 59 | 36+5 | 4.17 |
| BI-3 | M | 28+6 | 1280 | Bilateral frontotemporal subarachnoid space was slightly wider; Encephalomalacia around left ventricle with a small amount of bleeding; Transparent compartment cavity was full; The right cerebellar extraneous space was slightly wider. | Preeclampsia; RDS; HB; NEC; Pneumonia. | Yes | No | 47 | 35+4 | 11.64 |
| BI-4 | M | 28+4 | 1045 | Right frontotemporal encephalomalacia with compensatory dilation of right lateral ventricle | PPROM-7 days; RDS; HB; Sepsis. | Yes | No | 31 | 33+0 | 0.42 |
| BI-5 | M | 28+2 | 1400 | Multiple subependymal intracerebral hemorrhage and encephalomalacia around lateral ventricle; Bilateral ventricle dilation accompanied by bilateral ventricle occipital horn hematoma. | Triplet; RDS; HB; Brain hemorrhage and hydrocephalus; Inguinal hernia. | Yes | No | 26 | 32+0 | 0.52 |
| BI-6 | M | 29+1 | 1300 | Multiple abnormal cystic signals around bilateral ventricles-consider encephalomalacia. | severe asphyxia at birth; RDS; HB; Pneumonia; BPD. | Yes | No | 67 | 38+4 | 0.50 |
| BI-7 | M | 30+4 | 1950 | Multiple paraventricular echoes indicate the formation of encephalomalacia. | PPROM-2h; RDS; HB; Neonatal asphyxia; | Yes | No | 20 | 33+3 | 0.2 |
| BI-8 | M | 30+1 | 1370 | Encephalomalacia in bilateral frontal parietal lobes, around lateral ventricles and basal ganglia. | Threatened premature labor; RDS; Brain hemorrhage; Pulmonary hemorrhage; Pneumonia; BPD; | Yes | No | 36 | 35+2 | 1.33 |
| BI-9 | M | 25+3 | 900 | Extensive low density of the brain*. | Placental abruption; RDS; HB; BPD; Sepsis; Pneumonia~~;~~ | Yes | Yes | 53 | 33+0 | 0.34 |
| BI-10 | M | 31+4 | 1836 | Multiple infarcts and encephalomalacia in both cerebral hemispheres; The posterior part of falx cerebri and the tentorium of the cerebellum increased in density-subarachnoid hemorrhage properly happens. Old blood accumulation in the posterior horns of both lateral ventricles was slightly reduced. | Twins; Threatened preterm labor; RDS; Pulmonary hemorrhage; Brain hemorrhage and hydrocephalus; Neonatal infection. | Yes | Yes | 22 | 34+5 | 0.50 |
| BI-11 | M | 30+1 | 1630 | Right lateral ventricle body - anterior corner subependymal hemorrhage; Encephalomalacia around right lateral ventricle; Abnormal signals in the straight sinus should be noted for the possibility of venous thrombosis; Left thalamic punctate abnormality with limited diffusion; Right lateral ventricle dilated. | PPROM-8 days; RDS; HB; NEC; Sepsis; Meningitis; Brain hemorrhage; Pulmonary hemorrhage. | Yes | Yes | 45 | 36+4 | 12.12 |
| BI-12 | M | 26+6 | 950 | Small subependymal hemorrhage of bilateral ventricles, and encephalomalacia around right ventricle. | Placental abruption? RDS; Neonatal asphyxia; Pneumonia; NEC; BPD. | Yes | Yes | 64 | 36+0 | 1.69 |
| BI-13 | M | 29+1 | 1120 | Encephalomalacia around lateral ventricles. | PPROM-1 day; RDS; HB; Neonatal infection. | Yes | Yes | 42 | 35+1 | 0.20 |
| BI-14 | F | 31+6 | 1930 | Hemorrhage and encephalomalacia in left cerebellum. | Preeclampsia; RDS; HB. | No | Yes | 15 | 34+0 | 0.96 |
| BI-15 | M | 27+5 | 825 | Deep white matter injury and periventricular leukomalacia | PPROM-6 days; RDS; HB; Pulmonary hemorrhage; Sepsis; ROP (Right). | No | Yes | 45 | 34+2 | 10.67 |
| BI-16 | F | 28+3 | 1160 | Periventricular leukomalacia | PPROM-3 days; Twins; RDS; Pneumonia; BPD; Ileum perforation. | No | Yes | 69 | 38+2 | 4.68 |
| BI-17 | M | 31+6 | 1900 | Punctate hemorrhage and periventricular leukomalacia. | Twins; twin-to-twin transfusion syndrome; RDS; Neonatal asphyxia; Brain hemorrhage; ROP? | No | Yes | 18 | 34+3 | 5.21 |
| BI-18 | F | 31+5 | 1800 | Multiple encephalomalacia in both cerebral hemispheres. | Twins; One fetus died in utero; RDS; HB; Neonatal infection. | No | Yes | 24 | 35+1 | 5.37 |

*BI-9 brain imaging was conducted via computed tomography (CT) due to dyspnea of unknown origin at the time, primarily to assess lung tissue, with brain imaging performed concurrently. BI- Brain injury; NBI- No major brain injury. MRI- cerebral magnetic resonance imaging; PPROM- Preterm premature rupture of membranes. RDS- respiratory distress syndrome; HB- Hyperbilirubinemia; BPD- Bronchopulmonary dysplasia; NEC-Necrotizing enterocolitis; ROP- Retinopathy of prematurity. CRP: C-reactive protein.
